# Early Post-Transplant Leptin Concentration Changes in Kidney Transplant Recipients

**DOI:** 10.3390/medicina57080834

**Published:** 2021-08-17

**Authors:** Diana Sukackiene, Agne Laucyte-Cibulskiene, Ignas Badaras, Laurynas Rimsevicius, Valdas Banys, Dalius Vitkus, Marius Miglinas

**Affiliations:** 1 Institute of Clinical Medicine, Faculty of Medicine, Vilnius University, 01513 Vilnius, Lithuania; agne.laucyte@gmail.com (A.L.-C.); i.badaras97@gmail.com (I.B.); laurynas.rimsevicius@santa.lt (L.R.); marius.miglinas@santa.lt (M.M.); 2 Institute of Biomedical Sciences, Faculty of Medicine, Vilnius University, 01513 Vilnius, Lithuania; valdas.banys@santa.lt (V.B.); dalius.vitkus@santa.lt (D.V.)

**Keywords:** leptin, malnutrition, kidney transplantation, bioelectrical impedance analysis

## Abstract

*Background and Objectives*: Kidney transplant recipients represent a unique population with metabolic abnormalities, altered nutritional and immune status, as well as an imbalanced regulation of adipocytokine metabolism. Leptin is a hormonally active protein mainly produced by fat tissue that modulates appetite, satiety, and influences growth, energy, and bone metabolism. There has been great interest in the role of this hormone in chronic kidney disease-related protein energy wasting; thus, a positive leptin correlation with body mass index and fat mass was confirmed. This study was designed to determine the association of pre and post-kidney transplant leptin concentration with nutritional status and body composition. *Materials and Methods*: We studied 65 kidney transplant recipients. Nutritional status was evaluated before kidney transplantation and 6 months later using three different malnutrition screening tools (Subjective Global Assessment Scale (SGA), Malnutrition Inflammation Score (MIS), and Geriatric Nutritional Risk Index (GNRI)), anthropometric measurements, and body composition (bioelectrical impedance analysis (BIA)). Demographic profile, serum leptin levels, and other biochemical nutritional markers were collected. Statistical analysis was performed with R software. *Results*: Median age of the studied patients was 45 years, 42% were females, and 12% had diabetes. Leptin change was associated with body weight (*p* < 0.001), waist circumference (*p* < 0.001), fat mass (*p* < 0.001) and body fat percentage (*p* < 0.001), decrease in parathyroid hormone (PTH) (*p* < 0.001) transferrin (*p* < 0.001), diabetes mellitus (*p* = 0.010), and residual renal function (*p* = 0.039), but not dependent on dialysis vintage, estimated glomerular filtration rate (eGFR), or delayed graft function at any time during the study. After adjustment for age and sex, body mass index (BMI) (*p* < 0.001), fat mass (*p* < 0.001), and body fat percentage (*p* < 0.001) were independent variables significantly associated with post-transplant leptin change. Lower leptin values were found both before and after kidney transplantation in the SGA B group. GNRI as a nutritional status tool was strongly positively related to changes in leptin within the 6-month follow-up period. *Conclusions*: Kidney transplant recipients experience change in leptin concentration mainly due to an increase in fat mass and loss of muscle mass. GNRI score as compared to SGA or MIS score identifies patients in whom leptin concentration is increasing alongside an accumulation of fat and decreasing muscle mass. Leptin concentration evaluation in combination with BIA, handgrip strength measurement, and GNRI assessment are tools of importance in defining nutrition status in the early post-kidney transplant period.

## 1. Introduction

Kidney transplantation is a preferred renal replacement therapy for end-stage kidney disease (ESKD), providing a better health-related quality of life [1] and long-term survival [2]. However, the transition from ESKD to transplantation and further to the post-transplant period causes metabolic stress due to hormonal changes, shifts in nutrient intake and energy wasting [3], the loss of anorectic factors, low physical activity, the immunosuppressive treatment, the immune response to the transplant, rejection episodes, impaired kidney function, and the activation of systemic inflammation [4]. Thus, nutrition status evaluation and early identification of malnutrition is of crucial importance. Despite recent advances in diagnosing post-kidney transplant metabolic and nutrition complications, there is no consensus of the optimal strategy. Body composition analysis along with biomarkers, e.g., leptin, seems to improve malnutrition identification in ESKD [5].

Leptin is one of the major adipokines, first described as a biomarker of obesity two decades ago. Expression of leptin in visceral adipose tissue modulates appetite and satiety centers in the hypothalamus, influencing growth, energy, and bone metabolism. Emerging evidence suggests that leptin production is not limited to adipocytes, showing that skeletal muscle together with bone cells are also responsible for that [6]. Moreover, leptin receptors have been identified in human skeletal muscle [7] and their numbers may be reduced alongside muscle mass in patients with sarcopenia [8].

Notably, serum leptin release occurs in the circadian cycle, reaching a peak at night and therefore a disruption of the circadian balance can indirectly affect leptin secretion, thermogenesis, and energy homeostasis [9,10]. As a pro-inflammatory cytokine, it is responsible for innate and adaptive immune responses, and contributes to the generation and maintenance of low-grade inflammation that makes obese individuals more susceptible to develop cardiovascular diseases, type 2 diabetes, or degenerative [11,12,13] and autoimmune diseases [14,15]. 

Previous studies highlighted an important role of the kidney as the major site of leptin removal from the circulation. Renal uptake of circulating leptin in normal kidney functions as compared to kidney failure accounts for 12% and 0%, respectively [16]. Conversely, an increase in leptin synthesis takes place in the adipose tissue in CKD patients, which further contributes to hyperleptinemia. Chronic inflammation, hyperinsulinemia, and alterations in the fatty acid profile could be important factors that stimulate leptin synthesis in the adipose tissue in CKD [17].

In kidney transplant (KT) patients, leptin has been found to be associated with the estimated glomerular filtration rate (eGFR) [4] and graft survival [18], suggesting that lean individuals with lower leptin levels may benefit from kidney transplantation [19]. Studies that aimed to investigate the role of serum leptin in KTR are summarized in Supplemental Table S1. The association of leptin with nutritional parameters after kidney transplantation needs clarification and further investigation. Therefore, we aimed to determine the relationship of the leptin concentration with nutritional status and body composition during the early post-kidney transplant period. We hypothesized that leptin concentration six months post-kidney transplant, in regard to the baseline pretransplant value, plays an important role in diagnosing early post-transplant nutrition outcomes.

## 2. Subjects and Methods

### 2.1. Study Design and Patients

A follow-up observational study was conducted in a tertiary care university hospital between January 2018 and December 2019. The primary aim of this study was to evaluate the association of serum leptin level and nutritional status in the relevant KTR population, alongside patient demographics, biochemical markers, and bioelectrical impedance analysis (BIA) parameters. This study was approved by the regional research ethics committee (approval date 1 December 2017 and number 158200-17-972-470) and the research ethics committee of the hospital, and informed consent was obtained from all patients prior to enrollment in the study. Inclusion criteria were as follows: (1) undergoing hemodialysis (HD) > 3 months before KT; (2) age ≥ 18 years; and the (3) signed informed consent form. Exclusion criteria included patients with pacemakers; who were limbless; and patients who refused to participate in the study. 

### 2.2. Laboratory Data 

The blood samples were collected into BD Vacutainer SST-II Advance serum separator tubes (BD Diagnostics, Oxford, UK) by venipuncture after the overnight fast between 7:00 and 9:00 am. The samples for leptin and prealbumin were immediately centrifuged for 10 min at 1800 RCF (ambient temperature) and aliquoted serum was frozen at −20 °C until analysis. Samples for all other biochemical markers were centrifuged at the local clinical chemistry laboratory (3230 RCF, ambient temperature, 7 min) and analyzed the same day. Laboratory analysis was performed before KT and 6 months later. The list of performed tests and their analytical methods are summarized in Table 1 below. 

### 2.3. Anthropometric Data

The same nephrologist performed all anthropometric measurements for all participants. Height and post-dialysis weight were measured using an automatic scale with a sensitivity of 0.1 cm and resolution of 0.1 kg. BMI was calculated as the ratio between weight and height in meters squared (kg/m^2^). Waist and hip circumferences in centimeters were measured using a measuring tape and the waist-to-hip ratio was calculated.

### 2.4. Handgrip Strength 

Handgrip strength (HGS) was evaluated using ahydraulic hand dynamometer (model SH5002, SAEHAN Corp.,Changwon, South Korea) with a scale of strength of up to 100 kg. Maximum strength of the non-fistula dialysis arm or, if there was no fistula, on the dominant arm (because the arteriovenous fistula (AVF) is usually located in the non-dominant arm) was measured three times with an interval of 5 s between measurements and the highest recorded value was considered maximal grip strength. 

### 2.5. Evaluation of Nutritional Status

Body composition including intracellular water (ICW), extracellular water (ECW), total body water (TBW), fat free mass (FFM), skeletal lean mass (SLM), skeletal muscle mass (SMM), body cell mass (BCM), bone mineral content (BMC), and phase angle (PhA) were estimated by multiple frequency BIA. Measurements were performed by using the portable body bioimpedance spectroscopy device InBody S10 (Biospace, Seoul, Korea). Fat mass index (FMI) and lean mass index (LMI) were calculated as the quotient of fat mass/height^2^ and lean mass/height^2^ (kg/m^2^).

As there is still no specific or validated nutrition screening tool for chronic kidney disease, we have chosen to use the Subjective Global Assessment Scale (SGA) and Malnutrition Inflammation Score (MIS) that were administered through face-to-face interviews and have been previously reported to be applicable tools in this population [20].

The Geriatric Nutritional Risk Index (GNRI) calculated from the serum albumin level and the ratio between ideal and actual body weight. GNRI does not require patient interviewing and therefore it was chosen as an additional nutrition measure. It has been shown to be an appropriate nutrition screening tool and prognostic predictor in the CKD population [21].

### 2.6. Statistical Analysis

The results were expressed as the mean ± SD and median values. Data were tested for normal distribution by Shapiro–Wilk statistics. Categorical variables were analyzed by a Chi-square test. Comparisons between patients were performed using the Student’s *t*-test for normally distributed data or two–sample Wilcoxon test for non-parametric variables. Correlations of clinical parameters with serum leptin concentrations were evaluated by Pearson’s correlation test.

Leptin concentration change ‘∆leptin’ was calculated as: post-transplant leptin (6 months after kidney transplantation) concentration minus pre-transplant leptin concentration. Furthermore, multivariate forward stepwise regression analysis was conducted that evaluated the longitudinal change of ‘∆leptin’ adjusted for pre-transplant leptin concentration, age, sex, and its association with nutrition parameters and outcomes.

*p*-values lower than 0.05 were considered statistically significant. Statistical analysis was performed using 3.3.2 version R commander (Rcmdr, Canada).

## 3. Results

Out of 105 eligible recipients for deceased kidney transplantation (KTR), 65 subjects met the inclusion criteria and were enrolled in further analysis. Main pretransplant KTR characteristics are presented in Table 2. The most prevalent causes of ESKD were IgA nephritis and autosomal dominant polycystic kidney disease (16.9% and 13.8%, respectively). All patients received immunosuppressive treatment according to our local protocols including long-term immunosuppression with the calcineurin inhibitor, methylprednisolone, and mofetil mycophenolate. 

Significant leptin level correlations with pre and post-transplant variables are listed in Supplemental Table S2. eGFR wasn’t correlated to the leptin level (pre-transplant r = −0.013, *p* = 0.922; post-transplant r = 0.083, *p* = 0.514).

The comparison of pre-transplant and follow-up body composition measurement results, as well as nutritional and biochemical parameters, is presented in Table 3. Leptin levels significantly increased in 21 subjects and remained stable or decreased in others after the first 6 months post-kidney transplant. Although the post-transplant weight increase was documented, the BMI change was insignificant. According to BIA measurements, the decreased muscle mass and therefore weaker HGS was replaced by a striking increase in fat mass. Beyond previously mentioned changes, higher post-transplant transferrin, lower prealbumin, leptin, and PTH were observed, while other biochemical markers remained stable. Significantly lower pre and post-transplant leptin values were measured in the SGA B group.

‘∆Leptin’ (Table 3) was significantly associated with body weight, waist circumference, fat mass, body fat percentage, and HGS. Decreased PTH alongside a leptin decrease was observed. Other biomarkers failed to be associated with leptin. Worthy to note, diabetes mellitus (ß −6.773, *p* = 0.010) and residual renal function (coded as “no/yes”, ß 3.626, *p* = 0.039), but not dialysis vintage or delayed graft function, were associated with leptin change after renal transplantation. Interestingly, GNRI as a nutritional status tool was strongly positively related to changes in leptin within the 6 months follow-up period.

After conducting stepwise model selection using Bayesian information criterion (BIC), we identified independent variables significantly related to post-transplant leptin change (Table 4).

## 4. Discussion

Nutritional issues are highly relevant in all phases of kidney diseases and even in kidney transplantation. Wasting and frailty are related to negative outcomes in kidney transplant recipients; however, post-transplant diabetes, obesity, and accelerated atherosclerosis have a major impact on the overall prognosis [22]. Leptin evaluation is a promising biomarker for nutrition status assessment after kidney transplantation and calls for further assessments. Our report shows that in the early post-transplant period, leptin concentration change is mainly dependent on fat mass gain and decreasing handgrip strength. As HGS is a marker of muscle mass and alongside bioimpedance-derived evidence for muscle mass loss after kidney transplantation in study subjects, we suppose that leptin association with GNRI, but not SGA or MIS, could be explained by better GNRI performance in detecting sarcopenia [23,24].

We observed leptin concentration changes in concordance with a body composition shift, i.e., increase in visceral adiposity and fat mass, and with certain hormonal normalization, a decrease of PTH. These findings are in agreement with the studies of many other authors who clarified that leptin is mainly secreted into circulation from the adipose tissue and its concentration is positively associated with fat mass in both lean and obese individuals [25]. Furthermore, secondary hyperparathyroidism was associated with higher BMI as shown in patients with CKD stages 2–5 [26]. Data from experimental studies disagree on the relation between serum leptin and PTH [27]. Our findings were inconsistent with earlier epidemiological studies that showed a negative correlation of the parathyroid hormone and leptin in CKD, and higher leptin levels have been associated with reduced bone turnover [11]. Further studies are warranted for elucidating the potential pathophysiologic pathways between leptin and PTH. 

Kidney transplant recipients have usually a better nutritional status compared to dialysis patients not on a transplant waiting list. Based on all of the three used nutrition assessment tools (SGA, GNRI, and MIS), our patients had normal nutrition status or mild–moderate malnutrition. Only GNRI was related to leptin change largely due to the main constituents of this index, i.e., albumin and body weight, and due to the better performance in detecting sarcopenia [21]. Furthermore, elevated pre and post-transplant leptin concentration correlated with weaker handgrip strength. However, the handgrip strength decrease during the follow-up was associated with a simultaneous leptin decrease, possibly reflecting muscle wasting. The relationship between leptin and muscle mass is rather controversial. Previous studies have proved the release of leptin from skeletal muscle [28] and the presence of leptin receptors in both skeletal muscle and bone-derived mesenchymal (stromal) stem cells. These findings suggest that leptin can be an important part of a muscle–bone crosstalk [6]. Hubbard et al. reported low serum leptin levels in relation to muscle atrophy in the frailest older population, suggesting that aberrant leptin signaling is likely to play a significant role in sarcopenia [29]. 

Immunosuppression regimen that includes the administering of glucocorticoids (16 mg peroral methylprednisolone administration) could also be a significant factor in our cohort. Glucocorticoids play an important role in metabolic stress, especially in the early post-transplant period when doses are higher. They induce leptin mRNA in adipose tissues and lead to increased plasma levels of insulin, another leptin-inducing agent [30,31]. Some studies [32] show that increased food intake after glucocorticosteroid intake was surprisingly related to increased leptin levels, suggesting resistance to leptin-determined satiety signals and particularly explaining the leptin concentration change after kidney transplantation. 

Limitations of our study include the small sample size, the absence of a sex disaggregated analysis, and a case-matched control group. The main strengths include the fact that: this is an ongoing follow-up study analyzing the value of leptin concentration as an additional tool in the post-transplant nutrition status evaluation and our study used different nutrition assessment tools including questionnaires and bioelectrical impedance analysis.

## 5. Conclusions

We confirm that ESKD patients undergoing kidney transplantation obtain early post-transplant changes in leptin concentration (adjusted to age and sex) mainly due to the shift in body composition, i.e., increase in fat mass and loss of muscle mass. The GNRI score as compared to the SGA or MIS score identifies patients in whom the leptin concentration is increasing alongside the accumulation of fat and decreasing muscle mass. Hence, we suggest that leptin concentration evaluation in combination with BIA, HGS measurement, and GNRI assessment are tools of importance in defining nutrition status in the early post-kidney transplant period. Further research elucidating the leptin interplay with muscle mass, hormonal changes, and nutrition after kidney transplantation is warranted.

## Figures and Tables

**Table 1:** Analytical methods of the study.

Variable, Units	Method Details
Leptin, ng/mL	ELISA (IBL International GmbH, Hamburg, Germany) on Gemini analyzer (Stratec Biomedical, Bielefeld, Germany)
Albumin, g/L	Bromocresol green, colorimetric (Architect ci8200, Abbott, Chicago, IL, USA)
Prealbumin, g/L	Immunonephelometric (BN II, Siemens Healthineers, Erlangen, Germany)
Ferritin, µg/L	CMIA (Architect ci8200, Abbott, Chicago, IL, USA)
Transferrin, g/L	Immunoturbidimetric (Architect ci8200, Abbott, Chicago, IL, USA)
hs-CRP, mg/L	Latex immunoturbidimetric (Architect ci8200, Abbott, Chicago, IL, USA)
Total cholesterol, mmol/L	Enzymatic, cholesterol oxidase/cholesterol esterase (Architect ci8200, Abbott, Chicago, IL, USA)
LDL cholesterol, mmol/L	Calculated by Friedewald formula, but if TG > 4.5 mmol/L, direct enzymatic colorimetric (Architect ci8200, Abbott, Chicago, IL, USA)
TG, mmol/L	Enzymatic, glycerol phosphate oxidase (Architect ci8200, Abbott, Chicago, IL, USA)
PTH, pmol/L	CMIA (Architect ci8200, Abbott, Chicago, IL, USA)
eGFR	Calculated by CKD-EPI formula: creatinine method–enzymatic (Architect ci8200, Abbott, Chicago, IL, USA)

Abbreviations: ELISA, enzyme-linked immunosorbent assay; CMIA, chemiluminescent magnetic microparticle immunoassay; hs-CRP, high sensitivity C reactive protein; LDL, low density lipoprotein; TG, triglycerides; PTH, parathyroid hormone; and eGFR, estimated glomerular filtration rate.

**Table 2:** Pretransplant characteristics of enrolled KTR.

Variable, Units	Baseline [min; max] or ± SD
*n*	65
Age, years	45 [23; 69]
Sex, % women	42 (27)
Diabetes, %	12 (8)
Dialysis vintage, months	20 [0; 204]
BMI, kg/m^2^	25.3 [18.3; 42.8]
Residual renal function, % of yes	52
Delayed graft function, % of yes	62
Ischemia time, minutes	870 [30; 1650]
Subjective global assessment scale: A, % and B %	61.5 38.5
Geriatric nutrition index	114 ± 10
Malnutrition-Inflammation Score	5 [1; 10]

Abbreviations: SD, standard deviation and BMI, body mass index.

**Table 3:** Comparison of pre and post-transplant variables in KTR.

Variable, Units	Baseline Value [min; max] or ± SD	After 6 Months [min; max] or ± SD	*p* Value
Weight, kg	67.5 ± 29.1	79.6 ± 19.1	<0.001
BMI, kg/m^2^	25.3 [18.3; 42.8]	25.6 [16.0; 36.2]	0.797
Waist circumference, cm	94 ± 15	97 ± 16	<0.001
Hip circumference, cm	101 [54.0; 124]	102.2 ± 10.5	0.613
Conicity index	1.28 ± 0.1	1.31 ±0.1	<0.001
GNRI	114 ± 10	116 ±10	0.100
MIS	5 [1; 10]	1 [0; 10]	<0.001
Muscle mass, kg	34.4 ± 9.4	31.7 ±7.9	<0.001
Fat free mass, kg	61.6 ± 15.6	57.9 ±13.3	<0.001
Fat mass, kg	17.9 ± 11.9	21.7 ±11.9	<0.001
Body fat, %	21.7 ± 12.5	26.3 ±11.3	<0.001
Fat mass index	4.9 [0.5; 16.8]	6.3 [0.5; 17.2]	<0.001
HGS, kg	39 [15; 72]	32 [16; 63]	<0.001
Leptin, ng/mL	8.0 [0.02; 119.7]	5.81 [0.01; 45.63]	<0.001
Albumin, g/L	43.8 ± 3.6	44.6 ± 3.8	0.096
Prealbumin, g/L	0.429 ± 0.082	0.358 ± 0.074	<0.001
Ferritin, µg/L	304 [48; 914]	289 [19; 1819]	0.666
Transferrin, g/L	1.83 ± 0.28	2.17 ± 0.37	<0.001
hs-CRP, mg/L	1.9 [0.4; 24.7]	2.4 [0.1; 23.3]	0.324
Total cholesterol, mmol/L	5.88 [3.50; 11.85]	5.71 [3.94; 10.49]	0.696
LDL cholesterol, mmol/L	3.58 ± 1.23	3.56 ± 1.09	0.683
TG, mmol/L	2.15 [0.69; 6.13]	1.92 [0.80; 5.58]	0.734
PTH, pmol/L	87 [0.6; 212]	17 [0.7; 82]	<0.001

Abbreviations: BMI, body mass index; GNRI, geriatric nutritional risk index; MIS, malnutrition inflammation score; hs-CRP, high sensitivity C reactive protein; LDL, low density lipoprotein; TG, triglycerides; and PTH, parathyroid hormone.

**Table 4:** Leptin concentration change adjusted to pre-transplant leptin concentration, age and sex, and its influencing factors. Multivariate forward stepwise regression analysis.

	Multivariate Regression Analysis Models			Stepwise Model Selection (backward/forward)		
	ß	SE	*p*-Value	ß	SE	*p*-Value
∆Body weight, kg	0.151	0.067	0.028	-	-	-
∆BMI, kg/m^2^	1.725	0.375	<0.001	-	-	-
∆Waist circumference, cm	0.343	0.121	0.007	-	-	-
∆Fat mass, kg	0.420	0.120	<0.001	0.527	0.103	<0.001
∆Body fat, %	0.444	0.108	<0.001	-	-	-
∆PTH, pmol/L	0.115	0.056	0.045	0.129	0.042	0.004
∆Handgrip strength, kg	0.272	0.130	0.040	0.403	0.104	<0.001
∆Geriatric nutritional risk index	0.442	0.133	0.002	-	-	-

Only statistically significant models are listed above. All the ∆ are adjusted to pretransplant variable values. Abbreviations: ß—a standardized regression coefficient, BMI, body mass index and PTH, parathyroid hormone.

## Data Availability

The data presented in this study are available on request from the corresponding author. The data are not publicly available due to ethical restriction.

## Supplementary Materials

The following are available online at https://www.mdpi.com/article/10.3390/medicina57080834/s1.

## References

[1] Jansz T.T., Bonenkamp A.A., Boereboom F.T.J., Van Reekum F.E., Verhaar M., Van Jaarsveld B.C. (2018). Health-related quality of life compared between kidney transplantation and nocturnal hemodialysis. PLoS ONE.

[2] Kaballo M.A., Canney M., O’Kelly P., Williams Y., O’Seaghdha C.M., Conlon P.J. (2018). A comparative analysis of survival of patients on dialysis and after kidney transplantation. Clin. Kidney J..

[3] Park C.S., Lee S.-E., Cho H.-J., Kim Y.-J., Kang H.-J., Oh B.-H., Lee H.-Y. (2018). Body fluid status assessment by bio-impedance analysis in patients presenting to the emergency department with dyspnea. Korean J. Intern. Med..

[4] Małgorzewicz S., Dębska-Slizień A., Czajka B., Owczarzak A., Rutkowski B. (2016). Influence of Body Mass on Kidney Graft Function in Patients After Kidney Transplantation. Transplant. Proc..

[5] Yilmaz A., Kayardi M., Icagasioglu S., Canaan F., Gültekin F., Nur N. (2005). Relationship between Serum Leptin Levels and Body Composition and Markers of Malnutrition in Nondiabetic Patients on Peritoneal Dialysis or Hemodialysis. J. Chin. Med. Assoc..

[6] Hamrick M.W. (2017). Role of the Cytokine-like Hormone Leptin in Muscle-bone Crosstalk with Aging. J. Bone Metab..

[7] Guerra B., Santana A., Fuentes T., Delgado-Guerra S., Cabrera-Socorro A., Dorado C., Calbet J.A. (2007). Leptin receptors in human skeletal muscle. J. Appl. Physiol..

[8] Hamrick M.W., Herberg S., Arounleut P., He H.-Z., Shiver A., Qi R.-Q., Zhou L., Isales C.M., Mi Q.-S. (2010). The adipokine leptin increases skeletal muscle mass and significantly alters skeletal muscle miRNA expression profile in aged mice. Biochem. Biophys. Res. Commun..

[9] Serin Y., Tek N.A. (2019). Effect of Circadian Rhythm on Metabolic Processes and the Regulation of Energy Balance. Ann. Nutr. Metab..

[10] Kettner N., Mayo S.A., Hua J., Lee C., Moore D.D., Fu L. (2015). Circadian Dysfunction Induces Leptin Resistance in Mice. Cell Metab..

[11] Nagy K., Nagaraju S.P., Rhee C.M., Mathe Z., Molnar M.Z. (2016). Adipocytokines in renal transplant recipients. Clin. Kidney J..

[12] Stern J.H., Rutkowski J., Scherer P.E. (2016). Adiponectin, Leptin, and Fatty Acids in the Maintenance of Metabolic Homeostasis through Adipose Tissue Crosstalk. Cell Metab..

[13] La Cava A. (2017). Leptin in inflammation and autoimmunity. Cytokine.

[14] Zhang Y., Chua S. (2017). Leptin Function and Regulation. Compr. Physiol..

[15] Perrini S. (2019). Leptin: A marker of renal injury. Intern. Emerg. Med..

[16] Kataoka H., Sharma K. (2006). Renal handling of adipokines. Contrib. Nephrol..

[17] Korczynska J., Czumaj A., Chmielewski M., Swierczynski J., Sledzinski T. (2021). The Causes and Potential Injurious Effects of Elevated Serum Leptin Levels in Chronic Kidney Disease Patients. Int. J. Mol. Sci..

[18] Fonseca I., Oliveira J.C., Santos J., Malheiro J., Martins L.S., Almeida M., Dias L., Pedroso S., Lobato L., Henriques A.C. (2015). Leptin and Adiponectin During the First Week After Kidney Transplantation: Biomarkers of Graft Dysfunction?. J. Metab..

[19] Vieira P., Bassi J., LaRocca R.A., Castoldi A., Burghos M., Lepique A.P., Quintana F.J., Araujo R., Basso A., Strom T.B. (2013). Leptin Modulates Allograft Survival by Favoring a Th2 and a Regulatory Immune Profile. Am. J. Transpl..

[20] Ikizler T.A., Burrowes J.D., Byham-Gray L.D., Campbell K.L., Carrero J.-J., Chan W., Fouque D., Friedman A.N., Ghaddar S., Goldstein-Fuchs D.J. (2020). KDOQI Clinical Practice Guideline for Nutrition in CKD: 2020 Update. Am. J. Kidney Dis..

[21] Lin T.-Y., Hung S.-C. (2019). Geriatric Nutritional Risk Index Is Associated with Unique Health Conditions and Clinical Outcomes in Chronic Kidney Disease Patients. Nutrients.

[22] Cupisti A., Avesani C.M., D’Alessandro C., Garibotto G. (2020). Nutritional management of kidney diseases: An unmet need in patient care. J. Nephrol..

[23] Rasheedy D., El-Kawaly W.H. (2020). The accuracy of the Geriatric Nutritional Risk Index in detecting frailty and sarcopenia in hospitalized older adults. Aging Clin. Exp. Res..

[24] Vettoretti S., Caldiroli L., Armelloni S., Ferrari C., Cesari M., Messa P. (2019). Sarcopenia is Associated with Malnutrition but Not with Systemic Inflammation in Older Persons with Advanced CKD. Nutrients.

[25] Rafieian-Kopaei M. (2013). Serum leptin in renal transplant patients. J. Ren. Inj. Prev..

[26] Kovesdy C.P., Molnar M.Z., Czira M.E., Rudas A., Ujszaszi A., Rosivall L., Szathmari M., Covic A., Keszei A., Beko G. (2010). Associations between Serum Leptin Level and Bone Turnover in Kidney Transplant Recipients. Clin. J. Am. Soc. Nephrol..

[27] Polyzos S.A., Duntas L., Bollerslev J. (2017). The intriguing connections of leptin to hyperparathyroidism. Endocrine.

[28] Wolsk E., Mygind H., Grøndahl T.S., Pedersen B.K., van Hall G. (2012). Human skeletal muscle releases leptin in vivo. Cytokine.

[29] Hubbard R.E., O’Mahony M.S., Calver B.L., Woodhouse K.W. (2008). Nutrition, Inflammation, and Leptin Levels in Aging and Frailty. J. Am. Geriatr. Soc..

[30] Berneis K., Vosmeer S., Keller U. (1996). Effects of glucocorticoids and of growth hormone on serum leptin concentrations in man. Eur. J. Endocrinol..

[31] Agras P., Baskin E., Saatci U., Colak T., Cengiz N., Kinik S., Isiklar I., Haberal A., Mert I., Haberal M. (2005). Relationship Between Leptin and Bone Mineral Density in Renal Transplant Recipients. Transplant. Proc..

[32] Udden J., Bjorntorp P., Arner P., Barkeling B., Meurling L., Rossner S. (2003). Effects of glucocorticoids on leptin levels and eating behaviour in women. J. Intern. Med..

