# Supplementary material for: Early Post-Transplant Leptin Concentration Changes in Kidney Transplant Recipients
Source: Medicina (Kaunas). 2021 Aug 17;57(8):834. doi: 10.3390/medicina57080834 (PMC8399685; doi:10.3390/medicina57080834)
Supplement: Supplementary file 1 [file medicina-57-00834-s001.zip › medicina-1327647-supplementary.pdf]

medicina MDPI

Supplemental

Table S1. The list of the studies investigating the role of serum leptin in KTR.

Author (year)	Sample size/study design	Mean age	Correlations	Findings
Muras-Szwedziak et al. (2019) [1]	25/ follow up 3mo KTR vs CKD	46.9 ± 11.8	Positive with CRP	The impact of physical activity on adipokines
Marchelek-Mysliwiec et al. (2019) [2]	56/ cross-sectional KTR vs HD vs control	55.0 ± 12.4	Positive with BMI, BF, Klotho protein; negative with adiponectin	KT is associated with the increase of leptin, FGF23 and Klotho proteins
Dedinská et al. (2018) [3]	70/ follow up 6mo	46.7 ± 11.1	Positive with TG, PTDM, AR	Leptin is associated with PTDM and AR
Małgorzewicz et al. (2016) [4]	183/cross-sectional	51.7 ± 13.6	Positive with BMI and BF %; negative with LBM, eGFR	Leptin correlated with BMI and BF%, LBM and eGFR
Fonseca et al. (2015) [5]	40/follow up 7 days	ND	Positive with BMI, gender, age, DGF	Graft function was a strong determinant of leptinemia
Małgorzewicz et al. (2014) [6]	80/cross-sectional	52.4 ± 14.0	Positive with duration of KTX, BMI and BF%; negative with SGA	Increased BMI, abdominal obesity, and high leptin concentration are aggravated by time after transplantation and deterioration of graft function
Shu et al. (2014) [7]	280/cross-sectional	43.5 ± 12.7	Positive with MS, gender, BM, WC, BMI, hypertension, Cr, glucose, HbA1c, TG, uric acid	KT recipients with MS were associated with significantly higher serum leptin levels
Rafieian-Kopaei et al. (2013) [8]	72/cross-sectional	44 ± 12	Positive with gender; negative with duration of KTX	An inverse association between serum leptin and duration of renal transplantation
Nicoletto et al. (2012) [9]	32/ follow up 5 years	41.5 ± 11.4	Positive with BMI, gender, BF%, HOMA	Leptin levels and HOMA decrease in the immediate post-transplant period and remain reduced for at least 1 year
Teplan et al. (2012) [10]	70/follow up 12mo	ND	Positive with BF	An increase of body fat was associated with leptin
Lee et al. (2010) [11]	55/cross-sectional	40 to 61 years	Positive with WC, BMI, BF, TG, hs-CRP), TSF, midAFA	Serum leptin concentration correlates positively with MS in KT recipients
Molnar et al. (2010) [12]	993/cross-sectional	51 ± 13	Positive with age, MIS; negative with eGFR	MIS correlated significantly with all measures of nutritional status
Kovesdy et al. (2010) [13]	979/cross-sectional	50.9 ± 12.8	Positive with PTH; negative with vitD	Higher leptin levels were associated with higher PTH and lower vitamin D levels
Souza et al. (2007) [14]	32/ follow up 12mo KTR vs control	41.5 ± 11.4	Positive with BF, HOMA	Pretransplant leptin levels reduced after KT
Agras et al. (2005) [15]	41/cross-sectional	16 to 55 years	Positive with BMI, BMD	Elevated leptin levels are associated with increased BMD in KT recipients
Malyszko et al. (2005) [16]	27/cross-sectional	30 to 67 years	Positive with BF%, trunk fat, LBM, Cr, urea	Leptin levels are associated with graft function and BF
El Haggan et al. (2004) [17]	41/follow up 6mo	43.7 ± 10.4	Positive with BF, BMI, CRP	Pretransplant leptin levels reduced KT
Kayacan et al. (2003) [18]	34/ follow up 6mo KTR vs control	29 ± 9	Positive with BF%, HOMA	HOMA and BF% were found to be associated with leptin level
Kagan et al. (2002) [19]	25/cross-sectional KTR vs HTR vs LTR	46.0 ± 2.6	Positive with BMI, gender, Cr, CrCl	Leptin shows correlation with gender, BMI, kidney function, insulin and cortisol levels
Baczkowska et al. (2000) [20]	28/follow up 12mo KTR vs control	39.2 ± 10.6	Positive with BMI	Body weight and BMI was associated with increased leptin level
Kokot et al. (1998) [21]	40/ follow up until discharge from the hospital	34.3 ± 1.6	Positive with BMI; negative with age	Leptin levels reduced after KT

Howard et al. (1997) [22]	45/ cross-sectional KTR vs HD vs PD vs control	25 to 70 years	Positive with BMI, gender	Hyperleptinemia contributes to the anorexia and poor nutritional status in ESRD

CRP – C reactive protein; BMI – body mass index; BF – body fat, FGF23 – fibroblast growth l delayed graft function; KT – kidney transplantation, KTR – kidney transplant recipient; HTR – heart transplant recipient; LTR – liver transplant recipient; HD – hemodialysis; PD – peritoneal dialysis; K – healthy controls; CKD – chronic kidney disease; SGA – Subjective Global Assessment; TG – triglycerides; PTDM – post-transplant diabetes mellitus; AR – acute rejection; HOMA – homeostasis model assessment; MS – metabolic syndrome; BM – body mass; WC – waist circumference; Cr – serum creatinine; CrCl – creatinine clearance; TSF – triceps skinfold, AFA – arm fat area; MIS – malnutrition inflammation score; PTH – parathyroid hormone, vitD – vitamin D; BMD – body mineral density, ESRD – end-stage renal disease; ND – no data.

Table S2. Correlation between various variables and leptin levels.

	Pre-transplant leptin concentration		Post-transplant (after 6 months) leptin concentration	
	r	p-value	r	p-value
Weight, kg	0.252	0.042	0.305	0.013
BMI, kg/m^2	0.564	<.001	0.601	<.001
WC, cm	0.357	0.003	0.462	0.001
Body fat, %	0.643	<.001	0.757	<.001
Fat mass, kg	0.614	<.001	0.703	<.001
Muscle mass, kg	-0.194	0.119	-0.197	0.114
Albumin, g/L	-0.218	0.080	-0.148	0.238
PTH, pmol/l	0.316	0.010	0.278	0.024
GNRI	0.407	0.008	0.551	<.001
MIS	-0.257	0.038	-0.150	0.232
HGS, kg	-0.312	0.011	-0.212	0.089

BMI – body mass index, WC – waist circumference, GNRI – geriatric nutritional risk index, MIS – malnutrition inflammation score, HGS – handgrip strength.

References

1. Muras-Szwedziak, K.; Masajtis-Zagajewska, A.; Pawłowicz, E.; Nowicki, M. Effects of a Structured Physical Activity Program on Serum Adipokines and Markers of Inflammation and Volume Overload in Kidney Transplant Recipients. *Ann. Transplant.* **2019**, *24*, 569–575, doi:10.12659/aot.917047.
2. Marchelek-Myśliwiec, M.; Dziedziejko, V.; Nowosiad-Magda, M.; Wiśniewska, M.; Safranow, K.; Pawlik, A.; Domański, L.; Dołęgowska, K.; Stępniewska, J.; Ciechanowski, K. Bone Metabolism Parameters in Hemodialysis Patients with Chronic Kidney Disease and in Patients after Kidney Transplantation. *Physiol. Res.* **2019**, *68*, 947–954, doi:10.33549/physiolres.934118.
3. Dedinská, I.; Mäčková, N.; Kantárová, D.; Kováčiková, L.; Graňák, K.; Ľudovít, L.; Miklušica, J.; Skálová, P.; Galajda, P.; Mokáň, M. Leptin—A new marker for development of post-transplant diabetes mellitus? *J. Diabetes Complicat.* **2018**, *32*, 863–869, doi:10.1016/j.jdiacomp.2018.07.002.
4. Małgorzewicz, S.; Dębska-Slizień, A.; Czajka, B.; Owczarzak, A.; Rutkowski, B. Influence of Body Mass on Kidney Graft Function in Patients After Kidney Transplantation. Transplant. Proc. 2016, 48, 1472–1476, doi:10.1016/j.transproceed.2015.12.137.
5. Fonseca I, Oliveira JC, Santos J, Malheiro J, Martins LS, Almeida M, et al. Leptin and adiponectin during the first week after kidney transplantation: Biomarkers of graft dysfunction? Metabolism. 2015;64(2):202–7.
6. Małgorzewicz, S.; Dębska-Ślizień, A.; Czajka, B.; Rutkowski, B. Adipokines and Nutritional Status in Kidney Transplant Recipients. *Transplant. Proc.* **2014**, *46*, 2622–2626, doi:10.1016/j.transproceed.2014.09.014.
7. Shu, K.-H.; Wu, M.-J.; Chen, C.-H.; Cheng, C.-H.; Yu, T.-M.; Chuang, Y.-W.; Huang, S.-T.; Tsai, S.-F.; Lo, Y.-C.; Weng, S.-C.; et al. Serum Adipokine Levels in Renal Transplant Recipients. *Transplant. Proc.* **2014**, *46*, 381–384, doi:10.1016/j.transproceed.2013.11.033.
8. Rafieian-Kopaei M, Nasri H. Serum leptin in renal transplant patients. J Ren Inj Prev. 2013;2(2):55–557.
9. Nicoletto, B.B.; Souza, G.C.; Gonçalves, L.F.; Costa, C.; Perry, I.S.; Manfro, R.C. Leptin, Insulin Resistance, and Metabolic Changes 5 Years After Renal Transplantation. *J. Ren. Nutr.* **2012**, *22*, 440–449, doi:10.1053/j.jrn.2011.09.003.
10. Teplan, V.; Malý, J.; Gürlich, R.; Kudla, M.; Pit'Ha, J.; Racek, J.; Haluzík, M.; Šenolt, L.; Stollova, M. Muscle and Fat Metabolism in Obesity After Kidney Transplantation: No Effect of Peritoneal Dialysis or Hemodialysis. *J. Ren. Nutr.* **2012**, *22*, 166–170, doi:10.1053/j.jrn.2011.10.016.
11. Lee, M.-C.; Lee, C.-J.; Ho, G.-J.; Lee, C.-C.; Shih, M.-H.; Chou, K.-C.; Hsu, B.-G. Hyperleptinemia positively correlated with metabolic syndrome in renal transplant recipients. *Clin. Transplant.* **2010,** *24*, E124–E129, doi:10.1111/j.1399-0012.2010.01215.x.
12. Molnar, M.Z.; Keszei, A.; Czira, M.E.; Rudas, A.; Ujszaszi, A.; Haromszeki, B.; Kosa, J.P.; Lakatos, P.; Sárváry, E.; Beko, G.; et al. Evaluation of the Malnutrition-Inflammation Score in Kidney Transplant Recipients. *Am. J. Kidney Dis.* **2010**, *56*, 102–111, doi:10.1053/j.ajkd.2010.02.350.

13. Kovesdy, C.P.; Molnar, M.Z.; Czira, M.E.; Rudas, A.; Ujszaszi, A.; Rosivall, L.; Szathmari, M.; Covic, A.; Keszei, A.; Beko, G.; et al. Associations between Serum Leptin Level and Bone Turnover in Kidney Transplant Recipients. Clin. J. Am. Soc. Nephrol. 2010, 5, 2297–2304, doi:10.2215/CJN.03520410.
14. Souza, G.; Costa, C.; Gonçalves, L.; Manfro, R. Leptin Serum Levels in the First Year Post-Renal Transplantation. *Transplant. Proc.* **2007**, *39*, 439–440, doi:10.1016/j.transproceed.2007.01.040.
15. Agras, P.; Baskin, E.; Saatci, U.; Colak, T.; Cengiz, N.; Kinik, S.; Isiklar, I.; Haberal, A.; Mert, I.; Haberal, M.; et al. Relationship Between Leptin and Bone Mineral Density in Renal Transplant Recipients. Transplant. Proc. 2005, 37, 3106–3108, doi:10.1016/j.transproceed.2005.08.027.
16. Malyszko, J.; Pawlak, K.; Konstantynowicz, J.; Wolczynski, S.; Kaczmarski, M.; Mysliwiec, M. Correlations Between Leptin, Body Composition, Bone Mineral Density, and Bone Metabolism in Kidney Transplant Recipients. *Transplant. Proc.* **2005**, *37*, 2151–2153, doi:10.1016/j.transproceed.2005.03.001.
17. El Haggan, W.; Chauveau, P.; Barthe, N.; Merville, P.; Potaux, L.; Aparicio, M. Serum leptin, body fat, and nutritional markers during the six months post-kidney transplantation. *J. Metab.* **2004**, *53*, 614–619, doi:10.1016/j.metabol.2003.10.034.
18. Kayacan, S.M.; Yildiz, A.; Kazancıoğlu, R.T.; Sahin, S.; Sever, M.S.; Ark, E. The changes in serum leptin, body fat mass and insulin resistance after renal transplantation. *Clin. Transplant*. **2003**, *17*, 63–68, doi:10.1034/j.1399-0012.2003.02078.x.
19. Kagan, A.; Haran, N.; Leschinsky, L.; Sarafian, R.; Aravot, D.; Dolberg, J.; Ben-Ary, Z.; Rapoport, J. Serum Concentrations of Leptin in Heart, Liver and Kidney Transplant Recipients. *Isr. Med. Assoc. J.* **2002**, *4*, 213–217.
20. Baçzkowska, T.; Soin, J.; Soluch, L.; Lao, M.; Gaciong, Z. The role of leptin in body mass index increase in renal allograft recipients. *Transplant. Proc*. **2000**, *32*, 1331–1332.
21. Kokot, F.; Adamczak, M.; Wiecek, A. Plasma leptin concentration in kidney transplant patients during early post-transplant period. *Nephrol. Dial. Transplant.* **1998**, *13*, 2276–2280, doi:10.1093/ndt/13.9.2276.
22. Howard, J.K.; Lord, G.; Clutterbuck, E.J.; Ghatei, M.A.; Pusey, C.D.; Bloom, S.R. Plasma Immunoreactive Leptin Concentration in End-Stage Renal Disease. Clin. Sci. 1997, 93, 119–126, doi:10.1042/cs0930119.
